# A simple and rapid method for optical visualization and quantification of bacteria on textiles

**DOI:** 10.1038/srep39635

**Published:** 2016-12-22

**Authors:** Philipp Stiefel, Jana Schneider, Caroline Amberg, Katharina Maniura-Weber, Qun Ren

**Affiliations:** 1 Laboratory for Biointerfaces, Empa, Swiss Federal Laboratories for Materials Science and Technology, Lerchenfeldstrasse 5, CH-9014 St. Gallen, Switzerland; 2 Swissatest Testmaterialien AG, Mövenstrasse 12, CH-9015 St. Gallen, Switzerland

## Abstract

To prevent bacterial contamination on textiles and the associated undesired effects different biocidal coatings have been investigated and applied. However, due to health and environmental concerns anti-adhesive coatings preventing the binding of bacteria would be favored. To develop such anti-adhesive coatings simple assays for reliable and fast screening are beneficial. Here an easy-to-handle, robust and rapid assay to assess bacteria on textiles utilizing a tetrazolium salt was reported. The assay allowed direct eye visualization of the color change of the textiles containing bacteria, facilitating fast screening. Quantification of the adhered bacteria could be done by generating standard curves which correlate the staining intensity to cell numbers. An additional advantage of the described assay is that with the same detection method anti-adhesive and biocidal effects can be investigated. The method was applied to different coatings, using *Pseudomonas aeruginosa* and *Staphylococcus aureus* as model organisms. The detection limit was found to be between 2.5 * 10^6^ and 9.4 * 10^8^ for *P*. *aeruginosa* and between 1 * 10^6^ and 3.3 * 10^8^ for *S*. *aureus*. The anti-adhesive coating PLUMA was demonstrated to reduce bacterial adhesion without killing them, whereas the biocidal coating TH22-27 caused a clear reduction in the number of viable cells.

Clothes are often contaminated with bacteria during wearing and washing, which can lead to undesired effects such as unpleasant odor and damages of textile^1^,^2^,^3^,^4^. Some bacteria tightly stick to the textile and tend to form biofilm which is difficult to remove. Especially, polyester textiles which need to be washed at low temperatures are prone to rapidly develop malodor^5^,^6^,^7^. Additionally, contaminated textiles can cause health problems in the hospital environment. Therefore, many efforts have been dedicated to develop anti-bacterial textiles. Most coatings make use of biocidal substances, which kill bacteria^8^,^9^,^10^,^11^, but raise environmental and health concerns. More attractive approaches include usage of anti-adhesive textiles, which prevent the binding of bacteria^12^.

To develop new textile coatings a simple and fast method is desired to verify and evaluate the anti-bacterial and anti-adhesive efficacy. However, many existing quantification methods fail in properly detecting bacteria bound to textiles. Simple dyes such as crystal violet not only stain bacteria, but also textile fibers and result in high background signals. Staining with fluorescent dyes such as Syto9 is interfered by auto-fluorescence of most textiles^13^. Approaches which require releasing the bacteria prior to staining are imprecise as detachment by most methods is usually incomplete and biased by the coating. For the same reason plating of the released bacteria to determine colony forming units (CFU) is also inaccurate. Counting bacteria on microscopy images can be precise but is very time consuming and difficult due to different focal planes of the 3-dimensional structures of textile meshes. An alternative is to quantify viable bacteria by detection of metabolic activity such as measuring ATP and NADH levels. However, sophisticated detection devices are needed and the required substances such as luciferase/luciferin are often expensive.

Here we describe a simple and cost-effective method to quantify bacteria on textiles based on optical visualization using a tetrazolium salt. Iodonitrotetrazolium chloride (INT) was selected because it can change color from almost colorless to red/purple upon capturing two electrons. Electron sources are mainly ubiquinone and cytochrome of the respiratory chain of bacteria and the coenzyme NADH^14^,^15^. The purple compound is insoluble in water and forms crystals on the location where the INT captures electrons. These crystals were found to bind to the bacteria contaminated textile and could be solubilized with dimethylsulfoxid (DMSO) for quantification at an optical density of 490 nm (OD_490_). The method was applied to determine the numbers of bacteria bound to textiles with anti-adhesive coatings. Additionally, the killing effect of biocidal coatings was investigated. The staining intensities were correlated to the cell numbers by applying bacterial suspensions with different optical densities at 600 nm (OD_600_) onto textiles to generate a standard curve, which displayed a linear increase of staining intensity with the increase of the bacterial cell numbers. An anti-adhesive effect without being biocidal was observed for the PEG-based PLUMA coating, whereas a strong biocidal activity was found for the zinc pyrithione based coating TH22-27.

## Results

### Suitability of using tetrazolium salt for quantification of bacteria on textiles

To detect bacteria bound to textile, iodonitrotetrazolium chloride (INT) was selected. INT exhibits almost no color in water (transparent pale yellow solution) and is converted to a purple colored formazan compound which formed crystals on the bacteria attached textile upon capturing electrons. Visible colorization of the bacteria contaminated textiles occurred after a few minutes and became more intense with time. In the absence of bacteria, interaction of INT with different textile materials (polyester, wool, cotton and polyamide) resulted in negligible signal intensities of less than 0.03 (data not shown).

**Figure 1:**
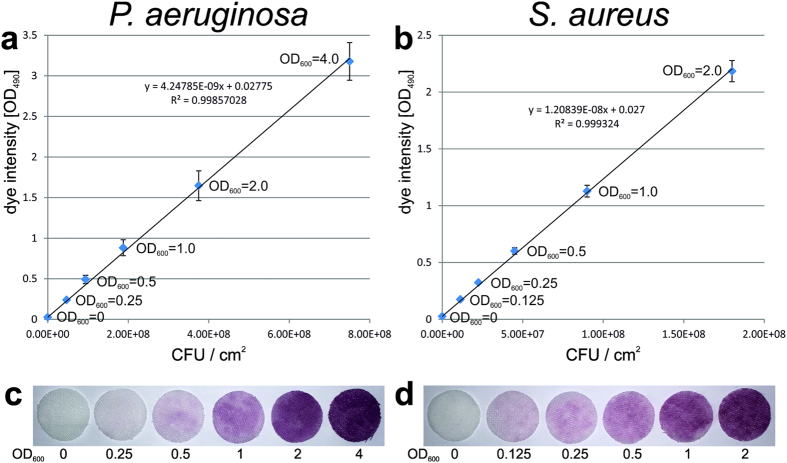
Standard curves of the INT staining intensity relative to the cell numbers. Solutions containing different numbers of *P*. *aeruginosa* (left) or *S*. *aureus* (right) cells were applied onto polyester textiles and stained with INT for 30 minutes. Bacteria CFU/cm^2^ (x-axis) are correlated to the intensity of INT staining at OD_490_ (y-axis). Initial OD_600_ of the applied bacterial solution is indicated for each data point. Appling a volume of 100 μl *P*. *aeruginosa* with an initial OD_600_ of 4 was found to correspond to 7.5 * 10^8^ bacteria per cm^2^ of textile after 1 hour incubation (a), while an OD_600_ of 2 equaled to 1.8 * 10^8^ *S*. *aureus* cells per cm^2^ (b). The value of background staining for the textiles incubated with the bacteria-free medium (OD_600_ = 0) was about 0.03. A linear regression (black line) through the background value precisely fits the staining intensity quantified for different cell numbers, indicating that there is no saturation of dye production in the tested cell densities. Error bars represent measurements for 4 individual textile samples.

To investigate whether the colorization of textiles by INT is in direct correlation with the number of attached bacterial cells, standard curves were generated using *P*. *aeruginosa* and *S*. *aureus* (Fig. 1). A linear increase of color intensity with the increase of viable cell numbers was observed for both bacterial species, and double of the bacterial cell numbers resulted in almost double of the signal intensity of the purple color (Fig. 1a,b). 50% differences in adhered cells within the tested range were clearly visible by eye (Fig. 1c,d).

### Assessment of anti-adhesive property of textiles

**Figure 2:**
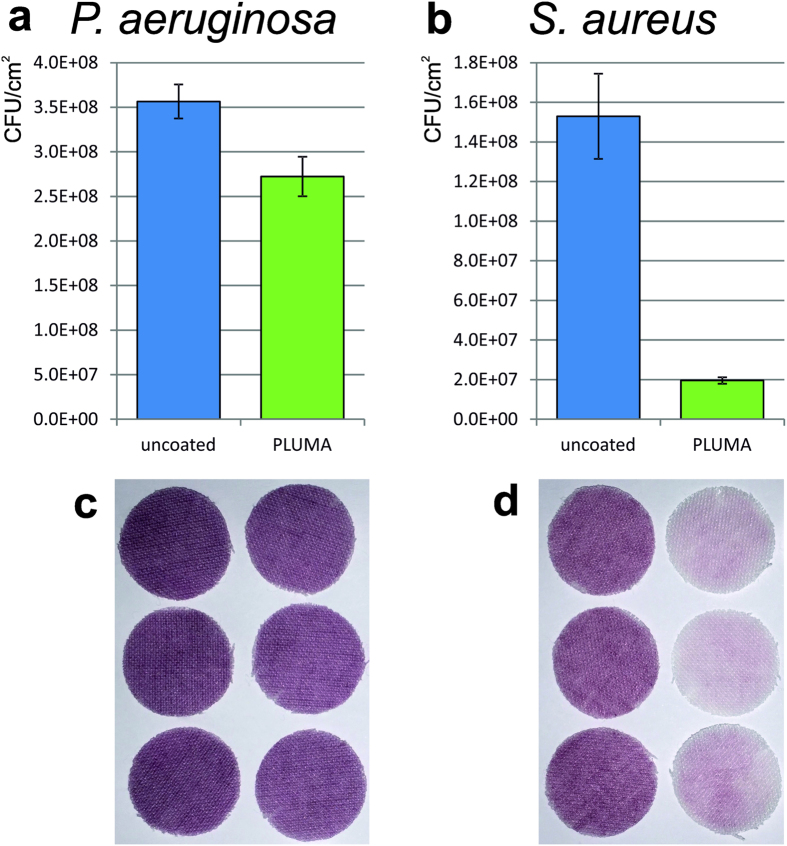
Assessment of anti-adhesive effect by the INT method. PLUMA coated polyester textiles (green) and the corresponding uncoated negative control (blue) were incubated in the same bacterial culture with an initial OD_600_ of 0.2. After washing 3 times vigorously, the textiles were stained with tetrazolium salt. PLUMA coating led to a reduction in the adhered cell numbers of 24% for *P*. *aeruginosa* (**a**) and 87% for *S*. *aureus* (**b**), respectively. The small difference in colorization for *P*. *aeruginosa* between the uncoated reference and the coated textile is recognized by eye with careful examination (**c**), while the difference for *S*. *aureus* is clearly visible (**d**). Error bars represent the standard deviation of 3 individual replicates.

A known anti-adhesive coating PLUMA was used to evaluate the developed INT method. PLUMA coated textiles showed 24% and 87% reduction in the numbers of adherent *P*. *aeruginosa* and *S*. *aureus* cells, respectively, compared to the corresponding uncoated control textiles (Fig. 2). Despite of the relative small difference (24%) between the coated and uncoated textiles for *P*. *aeruginosa*, it is possible to make a distinction in colorization by eye (Fig. 2c). The strong difference in binding of *S*. *aureus* to PLUMA coated textile is clearly visible in colorization (Fig. 2d).

**Figure 3:**
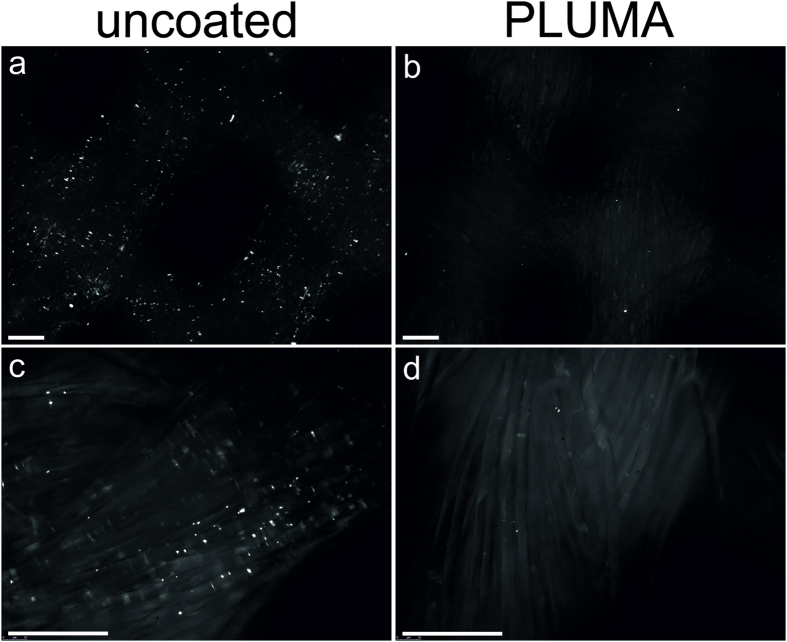
Microscopy analysis of SYTO9 stained textiles. The uncoated reference (left) and PLUMA coated (right) polyester textiles were incubated in the same culture of *S*. *aureus*. After washing 3 times vigorously, the textiles were stained with SYTO9. Green fluorescent images were taken using the 4× (a&b) and 20× (**c**,**d**) objective. The textile fibers exhibit slight background fluorescence, allowing the recognition of single and bundled fibers. The bacteria can be distinguished from the fibers as small intensive white spots on the images. The scale bars are 100 μm.

The textile samples were also stained with Syto9 and analyzed using fluorescence microscopy to visualize the attached bacterial cells. The cells could be readily distinguished from the fibers as small intensive spots. Uncoated reference textile was densely populated by *S*. *aureus* (Fig. 3a,c), while on the PLUMA coated textile only a few bacteria were observed (Fig. 3b,d). On the bacteria-free control textile no white spots were observed (data not shown). This confirmed the results obtained with INT method that PLUMA coating is anti-adhesive. However, the Syto9 microscopy method only allows semi-quantitative evaluation and conclusions on CFU per cm^2^ of textile are difficult because with the 3D structure of textiles just a fraction of the bacteria can be observed in the focal plane of the fluorescence microscope at one time.

### Assessment of biocidal property of textiles

**Figure 4:**
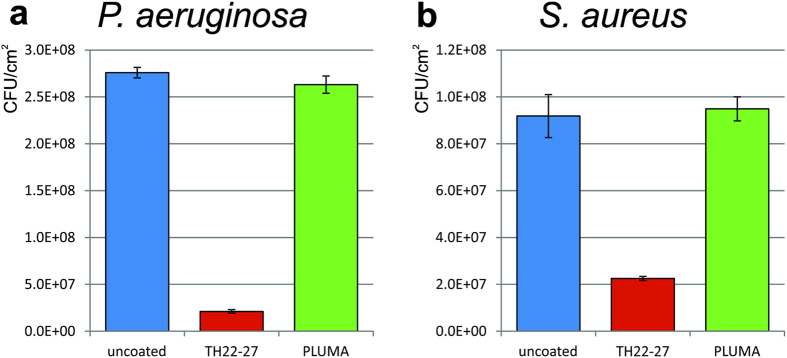
Biocidal activity of PLUMA and TH22-27 coatings. Culture solution of *P*. *aeruginosa* (**a**) or *S*. *aureus* (**b**) was directly added to the uncoated (blue), TH22-27 coated (red) and PLUMA coated (green) polyester textiles, respectively. After 1 hour incubation the bacteria contaminated textiles were stained with INT. The supernatant and the eluted dye were pooled in the same cuvette for quantification to avoid loss of the signal from the unbound cells, which was especially important for anti-adhesive coatings. Error bars represent the standard deviation of 3 individual replicates.

The INT detection method for quantification of bacteria was further evaluated for its suitability in testing biocidal coatings. The anti-adhesive PLUMA coating and a zinc pyrithione based biocidal coating (TH22-27) were tested against *P*. *aeruginosa* and *S*. *aureus* with modified experimental procedure to also include unbound cells, as described in Materials and Methods. For the PLUMA coating, no significant differences in cell viability was observed for both strains compared to the uncoated control textiles (Fig. 4), which was expected for an anti-adhesive coating. For the TH22-27 coating, 92% and 76% reduction in the viable cells were obtained for *P*. *aeruginosa* and *S*. *aureus*, respectively, compared to the uncoated textile (Fig. 4), demonstrating the biocidal activity of TH22-27. These results were confirmed by BacTiter-Glo assay (data not shown).

## Discussion

Up to now methods for quantification of bacteria bound to textiles have been rarely reported, especially those for the evaluation of anti-adhesive coatings^12^. Furthermore, existing methods are often based on complicated labelling and staining or require release of bacteria for culture-based procedures^12^,^16^,^17^,^18^. A simple and cost-effective method to quantify bacteria on textiles based on optical visualization was described here.

During the method development several tetrazolium salts were investigated for their ability to detect bacteria bound to textile. Iodnitrotetrazoliumchlorid (INT) was selected because it exhibits almost no color (light yellow) in solution and is converted to a water-insoluble purple formazan dye upon capturing two electrons from bacteria^14^. The resultant purple dye was found to form crystals which remained on the textile sample.

For different bacterial species the appropriate cell numbers and colorization time can be determined by adding bacterial cultures having different dilutions directly to the textile (Fig. 1). With about 4 * 10^8 ^cells/cm^2^ for *P*. *aeruginosa* and 1.5 * 10^8 ^cells/cm^2^ for *S*. *aureus*, visible purple color on the textile samples appeared after a few minutes and became very intense within 30 minutes. No visible colorization of the medium was observed, indicating that no significant amounts of bacteria and formazan crystals were released from the textile during the staining procedure. The purple formazan crystals could be easily dissolved from the samples using DMSO to allow the optical density measurement (Fig. S1). Thereby, a standard curve can be generated correlating the colorization of the textile (OD_490_) with the optical density of the applied bacterial culture (OD_600_) in a defined time frame. Corresponding cell numbers can be determined by plating the same culture. A linear increase of colorization with the increase of bacteria amount was observed and textiles without bacteria displayed almost no background signal (Fig. 1). The detection limit of the method was about 1 * 10^6^ and 2.5 * 10^6^ cells per cm^2^ for *S*. *aureus* and *P*. *aeruginosa*, respectively, allowing detection of more than 2 log reduction of viable cells. Even though the minimal detection limit of about 10^6^ is rather high, it is sufficient to allow rapid screening and detection of anti-adhesive or biocidal materials.

PLUMA coating is based on a PEGylated silane substance, which has similar side chain structure to PLL-*g*-PEG known for its anti-adhesive properties^19^. Applying the INT method to the textiles coated with PLUMA a clear reduction (87%) in the adhered cell number could be detected for *S*. *aureus*, but only slight reduction (24%) seen for *P*. *aeruginosa* (Fig. 2). Previously, it has been reported that most bacterial strains adhere poorly to polymer-brush coatings, with the exception of a *P*. *aeruginosa* strain^20^. It was postulated that cell surface hydrophobicity and surfactant release are the main factors promoting adhesion of *P*. *aeruginosa* strains to polymer-brush coatings^21^. This postulation can as well explain the observed higher adhesion of *P*. *aeruginosa* cells on PLUMA than *S*. *aureus* in this study.

To further verify that the effect of reduced INT signal for PLUMA coating is not caused by a biocidal effect (killing or attenuating the bacteria), bacteria were directly added to the textile and incubated for 1 hour without subsequent washing. No reduction in viable cells was observed (Fig. 4). Here it was important to include the supernatant for the quantification, because the supernatant contained unbound cells, which could lead to a significant INT signal. The results confirmed that the coating is not biocidal but reduces the number of adhered bacteria through an anti-adhesive effect.

Evaluation of biocidal coatings was first attempted in a setup similar to the adhesion assay, except that each textile type was incubated in a separate flask to avoid the cross effects of release toxic substances. Determined biocidal activities in this system were rather low probably due to the large volume used, causing a dilution effect of released biocidal substances. A system in which small amounts of bacteria suspension were directly added onto textiles was found to be more sensitive in detecting biocidal activity. The TH22-27 coating resulted in a clear reduction in the viable cells of *P*. *aeruginosa* and *S*. *aureus* (Fig. 4), which is expected for a zinc pyrithione based coating^22^,^23^,^24^. This confirms that a reduction in viable cells can be detected as decreased INT staining. The maximal detection limit was about OD_490_ 4, which corresponded to 3.3 * 10^8^ and 9.4 * 10^8^ cells per cm^2^ for *S*. *aureus* and *P*. *aeruginosa*, respectively; the minimal detection limit was about 0.03, which corresponded to 1 * 10^6^ and 2.5 * 10^6^ cells per cm^2^ for *S*. *aureus* and *P*. *aeruginosa*, respectively. Thus, the maximal reduction to be reliable detected is about 2 Log. A strong biocidal effect supposed to result in a more than 2 Log reduction of viable cells may not be distinguished from the weaker biocides. Therefore, the INT method is not suitable to detect strong biocidal effects of several log reduction. It is sensitive to small reductions and therefore rather useful to exclude biocidal effects than to quantify the activity of biocidal coatings.

In conclusion, the INT method described here is a fast and reliable method to quantify bacteria on textile materials. It allows optical assessment of bacterial adhesion by eye and a standard optical spectrometer is the only required instrument for quantification. This method does not encounter the problems that many other methods have such as high background signal, high cost and being time/labor intensive. The tetrazolium method neither requires release of the bacteria from surfaces nor microscopic observation and the dye does not display unspecific reactions with different textile materials and coatings. Another advantage of the INT method for detection of bacteria is that it can be used to evaluate both adhesion and biocidal effects. This allows to investigate the effect of anti-adhesive coatings and to exclude that they act as biocide with the same staining substance. The developed method can be envisaged to be applied for screening of other materials and coatings in addition to textile coatings tested in this study.

## Methods

### Chemicals and reagents

Chemicals and reagents were purchased from Sigma Aldrich (Switzerland) if not mentioned elsewise.

### Textiles

Polyester (Cat. No. 407), cotton (Cat. No. 221), wool (Cat. No. 217) and polyamide (Cat. No. 225) textiles were provided by Swissatest Testmaterialen AG, Switzerland. Before usage, the textiles were washed 3 times for 30 minutes at 60 °C in a washing machine without detergent. The uncoated, PLUMA (a PEGylated silane based) and TH22-27 (Zinc pyrithione based) coated polyester textiles were obtained from SANITIZED AG, Switzerland. PLUMA was applied at a concentration of 0.5% by Foullard technique. The coating consists of a 1:1 mixture (w/w) of (3-glycidyloxypropyl)trimethoxysilane and a PEGylated silane. The percent specifications indicate the weight amount of coating substance added per textile weight. For details see Patent WO2015091740 (PCT/EP2014/078383). Uncoated control textile was treated similarly without coating substances. Zinc pyrithione coating TH22-27 was applied at a concentration of 1% of textile weight. For experiments with bacteria round textiles with a diameter of 2 cm were punched using a metal stamper.

### Bacterial strains and growth conditions

*Pseudomonas aeruginosa* (DSM No. 1117) and *Staphylococcus aureus* (DSM No. 20231) were grown on Tryptic Soy Agar (TSA) at 37 °C. Liquid cultures were grown in 30% Tryptic Soy Broth (TSB, 9 g/l which corresponds to 30% of recommended concentration) supplemented with 2.5 g/l glucose at 37 °C and 160 rpm. Overnight cultures were diluted in 30% TSB supplemented with 2.5 g/l glucose and applied to 2 cm round textiles as follows.

### Standard curves to correlate the staining intensity with the numbers of bacterial cells

**Figure 5:**
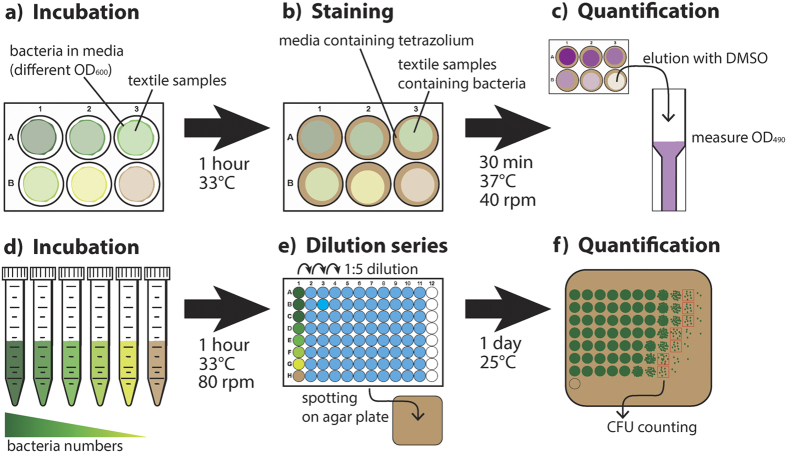
Scheme of the experimental procedure for generating the standard curve. Bacterial cells were directly added to the textiles and incubated to allow adhesion (**a**). The textiles were then stained with the medium containing INT (**b**). The dye was then eluted for quantification (**c**). The same culture applied to the textiles was also incubated in tubes (**d**) to prepare dilution series at the same time point as for staining (**e**), which were used to quantify bacterial CFU on agar (**f**). Using different dilution of bacterial culture allowed the generation of a standard curve correlating INT staining intensity with CFU.

For generating standard curve 100 μl culture of different concentrations between OD_600_ 0.125 and OD_600_ 4 was directly applied to the textile samples in 6-well plates (Fig. 5a). After 1 hour of incubation at 33 °C the samples were stained with tetrazolium salt (Fig. 5b). 3 ml of 30% TSB supplemented with 2.5 g/l glucose and 0.5 mg/ml 2-(4-Iodophenyl)-3-(4-nitrophenyl)-5-phenyl-2H-tetrazolium chloride (INT) was added to each textile. After incubating for 30 minutes at 37 °C and 40 rpm shaking, the liquid was removed and 1 ml dimethyl sulfoxide (DMSO) was added to each well. The dye was resolved by pipetting the DMSO several times onto the textile until a homogenous color was obtained. Absorption of the eluate was measured at 490 nm using DMSO as a blank (Fig. 5c). As a background control the different textiles were treated in the same way using media without bacteria. To properly correlate OD_600_ values of the applied culture with cell numbers, colony forming units (CFU) had to be determined at the same time point as the staining of bacterial culture. Therefore, the same bacterial solutions as added to textiles were also incubated for 1 hour at 33 °C and 80 rpm (Fig. 5d) and then used to determine cell numbers by plating a 1:5 dilution series on TSA (Fig. 5e). CFU of appropriate dilutions were counted after 1 day (Fig. 5f).

The bacterial culture with an OD_600_ value of 2.0 corresponded to about 4 * 10^8^ bacteria per square centimeter of textile for *P*. *aeruginosa* and 1.8 * 10^8^ bacteria per square centimeter for *S*. *aureus* (Fig. 1). These numbers were in a good range for quantification with INT and allowed the detection of reduced adhesion if a coating is applied to the textile (about 2 Log reduction).

### Quantification of bacterial adhesion on textiles

**Figure 6:**
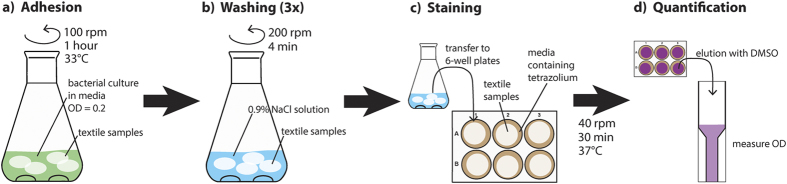
Scheme of the experimental procedure for the adhesion assay. Different textiles were incubated in the same bacterial culture (**a**), and subsequently washed vigorously to remove unbound cells (**b**). Textiles were then stained with INT (**c**) to quantify the amount of bacteria by comparing the signal intensity to the standard curve (**d**).

Incubation of the textiles in a 100 ml liquid culture having an initial OD_600_ of 0.2 for 1 hour displayed similar intensity of INT staining to that derived from direct addition of 100 μl culture having initial OD_600_ of 2.0 to the textile in the standard curve. Additionally, it was observed that the OD_600_ in the bacterial culture with textile was only slightly increased after 1 hour, while it increased much more in a similar culture without textiles. This demonstrates that bacterial cells favored to bind to the textiles than staying in suspension. Therefore, the bacterial culture was diluted to OD_600_ of 0.2 in 30% TSB supplemented with 0.25% glucose for the adhesion assay. For direct comparison different textile samples were incubated for 1 hour in the same baffled Erlenmeyer flask containing 100 ml of the bacterial culture at 33 °C. 100 rpm shaking was used to avoid that the textile samples stick to the bottom and to keep a homogenous bacterial culture (Fig. 6a). After the incubation the textiles were washed 3 times with 0.9% NaCl solution at 200 rpm shaking for 4 minutes to remove loosely bound bacteria (Fig. 6b). Textiles were then transferred individually to 6-well plates and 3 ml growth medium containing 0.5 mg/ml INT was added (Fig. 6c). During incubation at 37 °C and 40 rpm shaking the textiles accumulates purple color which can be eluted by 1 ml DMSO and quantified by measuring absorbance at OD490 nm (Fig. 6d). For background control the same procedure was done using medium without bacteria during adhesion, which resulted in a background staining of about 0.03 for coated as well as reference textiles.

### Quantification of bacterial viability on textiles

**Figure 7:**
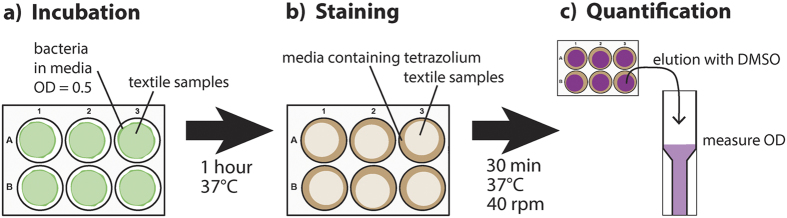
Scheme of the experimental procedure for the biocidal activity assay. Bacteria are directly added to the textile samples and incubated to allow killing by the biocide (**a**). The textiles were then stained with the medium containing INT (**b**). The amount of viable cells was quantified by comparing the measured signal intensity to the standard curve (**c**).

For analysis of biocidal properties of textile coatings, 200 μl bacterial culture having an OD600 of 0.5 in 30% TSB supplemented with 0.25% glucose was directly applied to the textile samples in 6-well plates and incubated for 1 hour at 37 °C (Fig. 7a). 0.5 ml growth medium containing 2 mg/ml INT was added to the textile without removing unbound bacteria (Fig. 7b). After incubation for 30 min at 37 °C and 40 rpm the dye was eluted from the textiles with 1 ml DMSO and pooled with the supernatant, in order not to lose the unbound bacteria. Staining intensity was quantified by absorbance measurement at 490 nm (Fig. 7c). For background control the same procedure was done using media without bacteria during incubation.

### Microscopy

Textile samples with bacteria were prepared and washed as described in the adhesion assay. For the staining 1 ml 2.5 μM SYTO9 (life technologies) in 0.9% NaCl solution was added per well. After 30 min of incubation the samples were placed into a Petri dish filled with 0.9% NaCl solution. Microscopy pictures were immediately taken using a 20× magnification water immersion objective (or 4× magnification air objective) and GFP filters with the Leica DM6000 B microscope, Leica DFC450 C camera and the Leica LAS AF software.

### BacTiter-Glo assay

The bacterial culture was diluted to OD_600_ of 0.5 in 30% TSB supplemented with 0.25% glucose. A volume of 50 μl bacterial culture was added to 7 mm diameter round textiles in 96-well plates. Plates were incubated for 1 hour at 37 °C. 100 μl 100% TSB was added per well. After 15 minutes of incubation at 37 °C, 100 μl BacTiter-Glo reagent (Promega), prepared according to the manufacturer’s instruction, were added per well. The plates were incubated for 5 min in the dark on an orbital shaker. The luminescence intensity was measured using Synergy HT Multi-Detection Microplate Reader (BioTek^®^) with gain 135 for 1 second per well.

### Detection limits

In this study, the minimal detection limit (the minimal reliable signal detected) is defined using IUPAC definition (http://goldbook.iupac.org/L03540.html, IUPAC Compendium of Chemical Terminology - the Gold Book) to enable clear discrimination from background noise: the signal should be larger than the background signal by three times of the standard deviation of the background^25^. Based on this definition and the results presented in Fig. 1, the minimal detection limit was found to be 1 * 10^6^ and 2.5 * 10^6^ cells per cm^2^ for *S*. *aureus* and *P*. *aeruginosa*, respectively. Furthermore, the maximal detection limit (the maximal reliable signal detected) was found to be 3.3 * 10^8^ and 9.4 * 10^8^ cells per cm^2^ for *S*. *aureus* and *P*. *aeruginosa*, respectively, based on the maximal optical density (OD_490_ of about 4) being reliably measured by the optical spectrometer.

### Statistical evaluation

For each sample the adhesion value was calculated by subtracting the value of textiles treated without bacteria from the arithmetic mean of 4 textiles with bacteria. Sample standard deviations were calculated from the values of the 4 similarly treated textiles. Three independent repeats were performed for each experiment.

## Additional Information

**How to cite this article**: Stiefel, P. *et al*. A simple and rapid method for optical visualization and quantification of bacteria on textiles. *Sci. Rep.* **6**, 39635; doi: 10.1038/srep39635 (2016).

**Publisher's note:** Springer Nature remains neutral with regard to jurisdictional claims in published maps and institutional affiliations.

## Supplementary Material

Supplementary Information
