# Supplementary material for: A simple and rapid method for optical visualization and quantification of bacteria on textiles
Source: Sci Rep. 2016 Dec 22;6:39635. doi: 10.1038/srep39635 (PMC5177907; doi:10.1038/srep39635)
Supplement: Supplementary Information [file srep39635-s1.pdf]

A simple and rapid method for optical visualization and quantification of bacteria on textiles

Philipp Stiefel[1], Jana Schneider[1], Caroline Amberg[2], Katharina Maniura-Weber[1], Qun Ren[1]*

[1] Laboratory for Biointerfaces, Empa, Swiss Federal Laboratories for Materials Science and Technology, Lerchenfeldstrasse 5, CH-9014 St. Gallen, Switzerland

[2] Swissatest Testmaterialien AG, Mövenstrasse 12, CH-9015 St. Gallen, Switzerland

* Corresponding author
E-mail: qun.ren@empa.ch

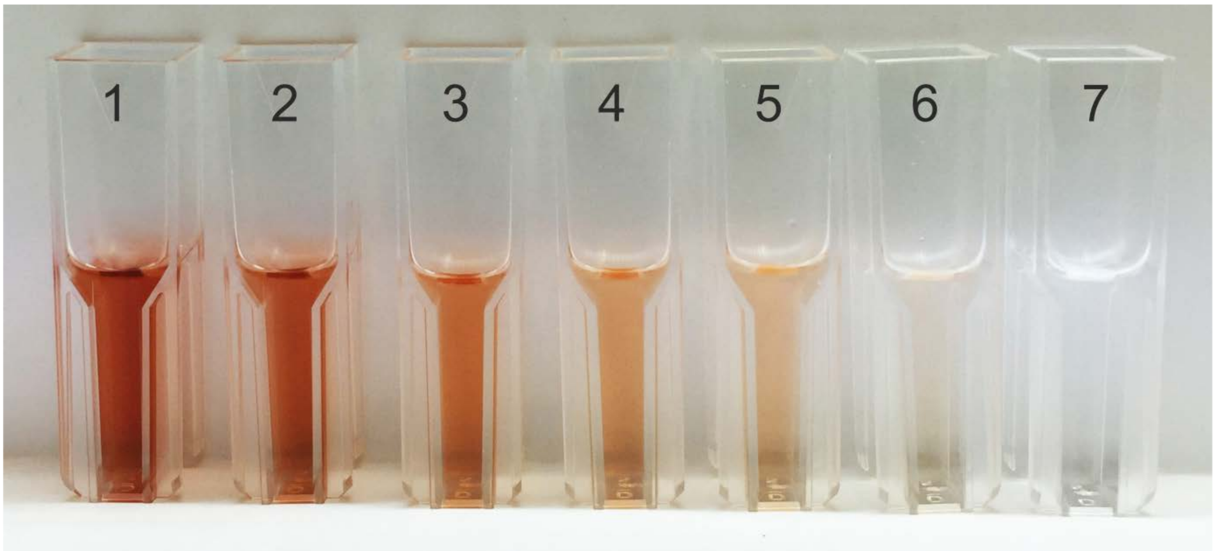

Figure S1: Eluted dye in cuvette. Dye eluted from textiles stained with INT after incubation with different numbers of bacteria (1-5) and medium w/o bacteria (6). DMSO blank (7) is also displayed.
